# Reliability of Manual Pulse Checks Versus Emerging Techniques in Pediatric Resuscitation: A Systematic Review

**DOI:** 10.7759/cureus.94765

**Published:** 2025-10-17

**Authors:** Mohammed Basher Ibrahim Alkhalifa, Omar Abbas, Nesreen Ahmed Taher Said Ahmed Khereldin, Baraah Mohammed Adham Ali, Lina Alghaly Alhabib Mohammed, Aala Hago Hamad Elneil Bakhit, Aya Bayoumy

**Affiliations:** 1 General Pediatrics, Neonatal Unit, Barnsley District General Hospital, South Yorkshire, GBR; 2 Neonatal Intensive Care Unit, Alhammadi Hospital, Riyadh, SAU; 3 Pediatrics, Neonatal Intensive Care Unit, Dubai London Hospital, Dubai, ARE; 4 Pediatrics, Pinderfields General Hospital, Wakefield, GBR; 5 Pediatrics, Manzil Healthcare, Abu Dhabi, ARE; 6 Pediatrics, Northampton General Hospital, Northampton, GBR

**Keywords:** cardiac arrest, diagnostic accuracy, manual palpation, pediatric resuscitation, pocus, point-of-care ultrasound, pulse check, systematic review

## Abstract

The accurate and rapid assessment of circulation is critical during pediatric cardiac arrest. Current guidelines recommend manual pulse checks, yet concerns remain regarding their reliability, as delays and inaccuracies may lead to inappropriate interruptions of cardiopulmonary resuscitation (CPR). Emerging techniques, such as point-of-care ultrasound (POCUS), offer potential objective alternatives. This systematic review aimed to synthesize the available evidence on the reliability of manual pulse checks compared with emerging techniques in pediatric resuscitation.

Following Preferred Reporting Items for Systematic Reviews and Meta-Analyses (PRISMA) 2020 guidelines, a systematic search of PubMed, Web of Science, Scopus, and Embase was conducted without date restrictions. Eligible studies compared manual pulse palpation with techniques such as POCUS or auscultation in pediatric populations, either in simulated or clinical settings. Data on study characteristics, diagnostic accuracy, time to decision, and clinical outcomes were extracted, and the risk of bias was assessed using the Quality Assessment of Diagnostic Accuracy Studies - Version 2 (QUADAS-2) tool. A narrative synthesis was performed due to heterogeneity among studies. Six studies met the inclusion criteria. Overall, the evidence indicated notable limitations of manual pulse checks, while POCUS demonstrated greater reliability and faster decision-making, in addition to providing supplementary diagnostic information such as confirmation of cardiac activity. However, most of the available studies were based on simulations or small case series, underscoring the need for larger prospective trials. Manual pulse palpation appears to be an unreliable method for assessing circulation in pediatric cardiac arrest. POCUS shows promise as a more accurate and efficient alternative, with potential to improve the quality of resuscitation. Further research in real-world clinical settings is warranted to establish its impact on survival and neurological outcomes.

## Introduction and background

Cardiac arrest in pediatric patients remains a critical emergency associated with high morbidity and mortality worldwide, with survival rates remaining markedly lower than in adults [1]. Timely and accurate recognition of circulatory status is central to effective resuscitation. Current pediatric advanced life support (PALS) and basic life support (BLS) guidelines emphasize rapid assessment of circulation through manual pulse palpation, typically at the carotid, femoral, or brachial arteries [2]. However, accumulating evidence questions the reliability of manual pulse checks, especially under high-stress resuscitation conditions [3]. Studies in both adult and pediatric populations have shown that manual palpation can be inconsistent and delayed, often resulting in false-positive or false-negative findings [4]. Such inaccuracies may cause unnecessary pauses or delays in chest compressions, potentially compromising survival and neurological outcomes [5].

To overcome these challenges, several emerging technologies, such as Doppler ultrasound, point-of-care echocardiography, near-infrared spectroscopy (NIRS), and capnography-based monitoring, have been explored as adjuncts or alternatives to manual pulse checks [6]. NIRS assesses tissue oxygenation by measuring changes in light absorption in the near-infrared range, providing continuous feedback on perfusion, while capnography measures end-tidal carbon dioxide (EtCO₂) as an indirect indicator of cardiac output and resuscitation quality [2]. These methods offer objective, real-time insights into circulation and may improve the speed and accuracy of assessments during pediatric resuscitation. Nevertheless, their clinical utility, feasibility, and impact on outcomes in pediatric populations are still being evaluated [6].

Given the essential role of prompt and accurate circulation assessment during pediatric cardiac arrest, a systematic review of the available evidence is warranted. This review aims to synthesize current data on the reliability of manual pulse checks compared with emerging techniques in pediatric resuscitation, focusing on accuracy, assessment time, feasibility, and clinical outcomes. By consolidating this evidence, it seeks to inform clinical practice and identify future directions that may enhance pediatric resuscitation protocols and outcomes.

## Review

Methodology

Protocol and Reporting

This systematic review was conducted in accordance with the Preferred Reporting Items for Systematic Reviews and Meta-Analyses (PRISMA) 2020 guidelines [7]. The review protocol was developed a priori to ensure methodological rigor and transparency, and all stages of the review process were guided by these principles.

Eligibility Criteria

We included original studies that assessed the reliability, accuracy, or feasibility of manual pulse checks compared with emerging techniques (e.g., ultrasound, Doppler devices, capnography, and NIRS) in the context of pediatric resuscitation. Studies were eligible if they involved pediatric populations (neonates, infants, children, and adolescents up to 18 years of age) undergoing simulated or real resuscitation scenarios. We included randomized controlled trials (RCTs), prospective and retrospective observational studies, and diagnostic accuracy studies. Studies focusing exclusively on adult populations, animal studies, conference abstracts without full text, and narrative reviews were excluded.

Information Sources and Search Strategy

A comprehensive literature search was performed in four major electronic databases: PubMed/MEDLINE, Web of Science, Scopus, and Embase (Elsevier). The search was conducted without any date restriction to capture the full scope of available evidence. The last search was completed on 02 September 2025. The search strategy combined controlled vocabulary (e.g., MeSH terms in PubMed and Emtree terms in Embase) and free-text keywords related to "pediatric resuscitation," "pulse check," "manual palpation," "ultrasound," "Doppler," "capnography," and "near-infrared spectroscopy." The detailed search strategies for each database are provided in Table 4 of the Appendices.

Study Selection

All identified records were imported into EndNote X6 reference management software (Thomson Reuters, Philadelphia, PA, USA), where duplicates were automatically and manually removed prior to screening. Two reviewers independently screened titles and abstracts for relevance. Full texts of potentially eligible studies were retrieved and reviewed against the predefined inclusion and exclusion criteria. Disagreements were resolved through discussion and consensus, with a third reviewer available if needed.

Data Extraction

A standardized data extraction form was used to collect information from each included study. Extracted data included study characteristics (author, year, country, and study design), participant details (age group, sample size, and clinical setting), intervention details (type of pulse assessment technique used), comparator (manual palpation), outcomes (accuracy, sensitivity, specificity, time to assessment, feasibility, and clinical impact), and key findings. Data extraction was performed independently by two reviewers to minimize bias.

Risk of Bias Assessment

The methodological quality and risk of bias of the included studies were assessed using the Quality Assessment of Diagnostic Accuracy Studies - Version 2 (QUADAS-2) tool [8], which is specifically designed for diagnostic accuracy studies. This tool evaluates the risk of bias across four key domains: patient selection, index test, reference standard, and flow and timing. Each domain was assessed for risk of bias and applicability concerns. Assessments were conducted independently by two reviewers, with consensus achieved through discussion.

Data Synthesis

Given the substantial heterogeneity in study designs, populations, settings, and outcome measures, a quantitative meta-analysis was not feasible. Studies differed in terms of the type of emerging technology evaluated, clinical versus simulated resuscitation contexts, and outcome definitions, precluding meaningful statistical pooling of results. Instead, we performed a narrative synthesis, structured around the comparison between manual pulse checks and individual emerging techniques. Key findings were summarized descriptively, with an emphasis on accuracy, time efficiency, feasibility, and clinical outcomes relevant to pediatric resuscitation.

Results

Study Selection and Characteristics

**Figure 1:**
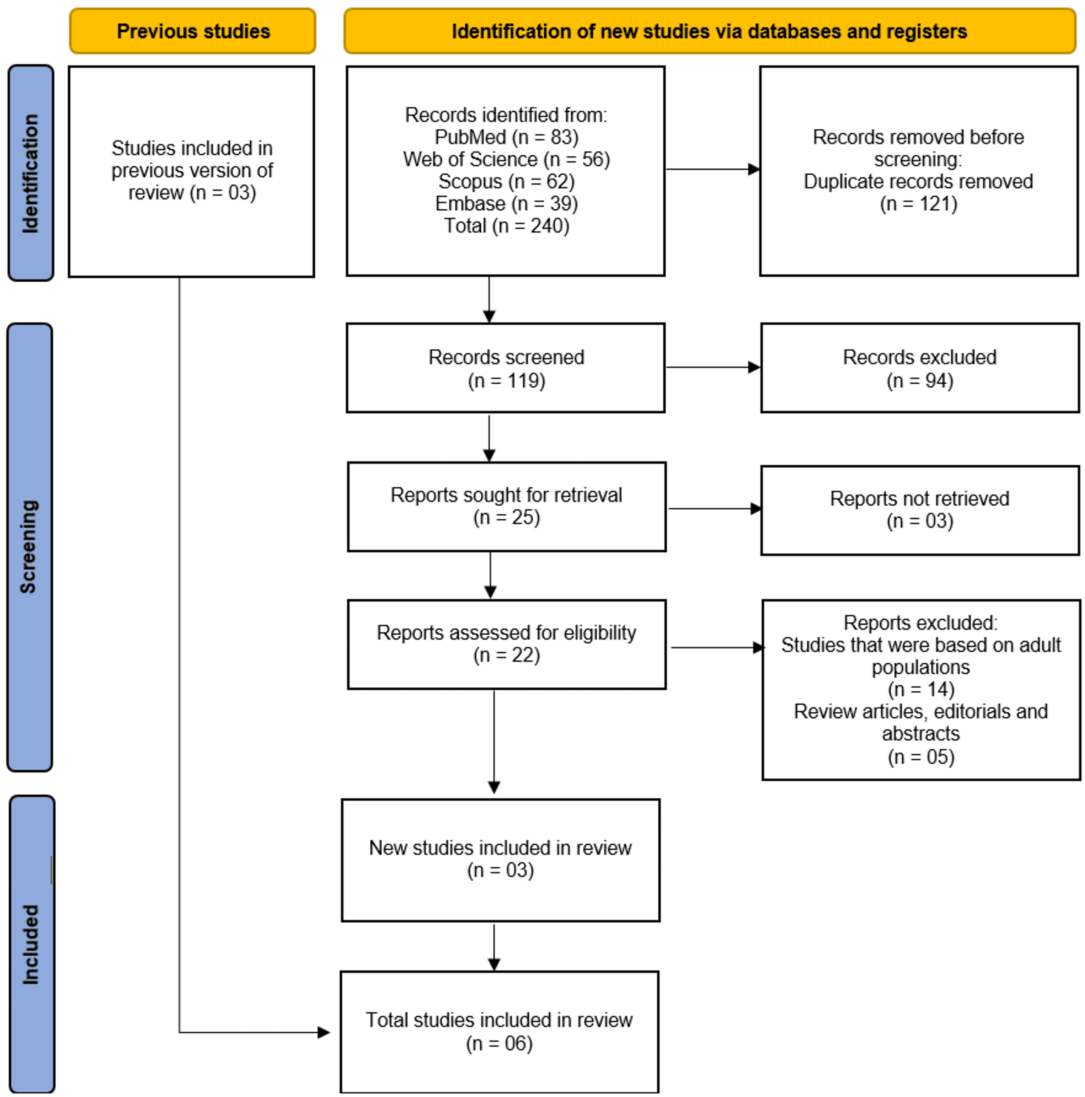
Studies identification

The systematic search across four electronic databases (PubMed, Web of Science, Scopus, and Embase) yielded a total of 240 records. After the removal of 121 duplicate records, 119 unique studies underwent initial title and abstract screening. Of these, 94 records were excluded as they did not meet the inclusion criteria. The full texts of the remaining 25 articles were sought for retrieval, of which three could not be obtained. Consequently, 22 reports underwent a detailed, full-text eligibility assessment. This process led to the exclusion of 19 articles, with 14 excluded for focusing on adult populations and five excluded for being review articles, editorials, or conference abstracts. Three new studies met the inclusion criteria and were added to the three studies carried forward from previous work [9], resulting in a total of six studies [10-15] being included in the final systematic review. The selection process is summarized in the PRISMA flow diagram (Figure 1).

The characteristics of these studies are summarized in Table 1. The included studies were published between 2003 and 2023 and were conducted in a range of countries, including Japan [10], Australia [11,12], and the United States [13-15]. The study designs were varied, encompassing experimental/observational studies [10,12], diagnostic accuracy/reliability studies [11], clinical observational studies and case series [13,14], and a cross-sectional survey [15]. The population focus was exclusively pediatric, with studies involving infants [10], children with an average age of 1.8 years [11], and a broader pediatric age range [12,13,15], as well as specific case reports [14]. The settings in which the studies were conducted included in-hospital environments (e.g., intensive care units and emergency departments) [11,13,14], simulation-based scenarios using manikins or video clips [10,12,15], and a combination of pre-hospital and in-hospital settings [14]. The techniques compared were primarily manual pulse palpation (assessing brachial, carotid, femoral, and apical sites) versus emerging techniques such as direct auscultation of the apical impulse [10] and various applications of point-of-care ultrasound (POCUS) [13-15].

**Table 1:** Characteristics of included studies VA-ECMO, venoarterial extracorporeal membrane oxygenation; LVAD, left ventricular assist device; POCUS, point-of-care ultrasound; IO, intraosseous; PEM, pediatric emergency medicine; ED, emergency department

Author (year)	Country	Study design	Population (age, N)	Setting (pre-hospital/in-hospital/simulation)	Technique(s) compared	Primary outcomes assessed
Inagawa et al. [10] (2003)	Japan	Experimental/observational study	Infants; 28 nurses performed assessments	Simulation (nurses detecting cardiac activity in infants)	Brachial pulse palpation, carotid pulse palpation, femoral pulse palpation, apical impulse palpation, direct auscultation of apical impulse	Time to detect pulse; accuracy of pulse detection within 10 seconds
Tibballs and Russell [11] (2009)	Australia	Diagnostic accuracy study (observer reliability study)	Infants and children, average age 1.8 years (range 1 week-13 years), N=209 rescuers (doctors and nurses) assessing 16 patients	In-hospital (patients on VA-ECMO or LVAD with nonpulsatile circulation)	Manual pulse palpation (brachial vs. femoral) vs. gold standard (investigators with cardiovascular data)	Accuracy, sensitivity, specificity of pulse palpation for diagnosing cardiac arrest
Tibballs and Weeranatna [12] (2010)	Australia	Observational/simulation-based study	17 children (1 day-11 years); 82 nurses, 71 doctors as rescuers	Simulation	Manual brachial pulse palpation	Accuracy and time to diagnose pediatric cardiac arrest; sensitivity and specificity; impact of rescuer experience
Tsung and Blaivas [13] (2008)	USA	Clinical observational/preliminary clinical observations	Pediatric patients in cardiac arrest	In-hospital (assumed, as point-of-care echocardiography is hospital-based)	Manual pulse check vs. focused point-of-care echocardiography	Correlation between pulse check and echocardiography; assessment of resuscitation effectiveness
Leviter et al. [14] (2023)	USA	Case series	4-month-old female (N=1), 12-year-old male (N=1)	Pre-hospital and in-hospital (pediatric ED)	Manual POCUS	Detection of cardiac activity, confirmation of IO access, guidance of resuscitation management decisions
Yanni et al. [15] (2023)	USA	Cross-sectional survey/case series	Pediatric patients (video clips), N=263 PEM attendings/fellows responding; primary subgroup N=110 experienced PEM attendings	Simulation (video-based survey of POCUS clips)	POCUS interpretation vs. cardiac standstill assessment	Interobserver agreement in identifying cardiac standstill (Krippendorff’s α)

Diagnostic Accuracy and Performance of Manual Pulse Palpation

The diagnostic performance of manual pulse palpation was evaluated across multiple studies and was consistently found to have significant limitations. In a simulation study on infants, Inagawa et al. [10] reported that the accuracy of pulse detection within 10 seconds varied considerably by palpation site: brachial pulse palpation was 73.1% accurate, carotid was 50% accurate, and femoral was only 42.9% accurate. This study also highlighted that auscultation was the fastest (2.4 seconds) and most accurate (100%) technique. Tibballs and Russell [11], in an in-hospital study of children with non-pulsatile circulation, found that manual pulse palpation had an accuracy of 77-78%, a sensitivity of 0.85-0.86, but a concerningly low specificity of 0.56-0.67. This low specificity led to a high rate of misdiagnosis, with healthcare personnel withholding compressions in 14% of cases where they were needed and performing unnecessary compressions in 36% of cases where they were not [11]. A subsequent simulation-based study by Tibballs and Weeranatna [12] confirmed these findings, reporting an overall accuracy of 78% for manual pulse palpation in diagnosing pediatric cardiac arrest, with a mean decision time of 30 seconds. They noted that faster decisions (under 10 seconds) were associated with higher accuracy and that performance was influenced by rescuer experience [12].

Performance and Utility of POCUS

In contrast to manual methods, the emerging technique of POCUS demonstrated potential for greater reliability in assessing cardiac activity during pediatric resuscitation. An early observational study by Tsung and Blaivas [13] suggested that focused POCUS could provide a more objective correlation for the traditional pulse check, potentially optimizing resuscitation efforts by confirming the presence or absence of cardiac activity. This utility was illustrated in a case series by Leviter et al. [14], where manual pulse checks failed to detect cardiac activity in two pediatric patients, while POCUS successfully identified cardiac motion, confirmed intraosseous (IO) access, and guided critical resuscitation management decisions. The diagnostic reliability of POCUS for identifying cardiac standstill was formally assessed by Yanni et al. [15] in a video-based survey of pediatric emergency medicine (PEM) physicians. They reported substantial interobserver agreement (Krippendorff’s α=0.740) among experienced PEM attendings for interpreting cardiac standstill, though agreement was lower in more complex scenarios with subtle wall motion [15]. The interpretation time was rapid, averaging six seconds per clip, indicating its feasibility in a time-critical arrest scenario [15].

Synthesis of Comparative Findings

The comparative diagnostic accuracy and performance metrics of the included studies are detailed in Table 2. Collectively, the evidence indicates that manual pulse palpation is prone to error, with variable accuracy and speed, often leading to critical mistakes in the decision to initiate or withhold chest compressions [10-12]. The integration of POCUS appears to address several of these limitations by providing a direct, visual assessment of cardiac activity. While the evidence for POCUS is still evolving and includes preliminary observations [13,14] and simulation-based assessments [15], it consistently points toward higher reliability and faster, more accurate decision-making compared to manual techniques. However, challenges with POCUS include the potential for misinterpretation in cases with minimal cardiac wall motion and the need for adequate training to obtain and interpret images correctly [15].

**Table 2:** Diagnostic accuracy and performance of pulse check methods NR, not reported; ICC, intraclass correlation coefficient; Kα, Krippendorff’s alpha; POCUS, point-of-care ultrasound; IO, intraosseous; ROSC, return of spontaneous circulation; PEM, pediatric emergency medicine; CPR, cardiopulmonary resuscitation; VA-ECMO, venoarterial extracorporeal membrane oxygenation; LVAD, left ventricular assist device

Author (year)	Technique assessed	Accuracy (% or sensitivity/specificity)	Time to decision (s)	Inter-rater reliability (Kappa/ICC)	Clinical outcomes/errors reported
Inagawa et al. [10] (2003)	Brachial, carotid, femoral, apical palpation vs. direct auscultation	Auscultation 100%; apical 75%; brachial 73.1%; carotid 50%; femoral 42.9%	Auscultation 2.4; apical 3.5; brachial 4.0; carotid 9.9; femoral 9.1	NR	Auscultation fastest and most accurate; palpation slower, less reliable, prone to missed detection
Tibballs and Russell [11] (2009)	Manual pulse palpation (brachial/femoral)	Accuracy 77-78%, sensitivity 0.85-0.86, specificity 0.56-0.67	NR	NR	Misdiagnosis 22%; withheld compressions 14%; unnecessary compressions 36%
Tibballs and Weeranatna [12] (2010)	Manual pulse palpation	Accuracy 78%, sensitivity 0.76, specificity 0.79	Mean 30±19	NR	Unreliable for cardiac arrest diagnosis; accuracy and speed depend on experience; faster decisions (<10 s) are more accurate
Tsung and Blaivas [13] (2008)	Manual pulse check vs. focused point-of-care echocardiography	Manual pulse unreliable; echocardiography preliminary correlation	NR	NR	Manual pulse may delay or produce inaccurate CPR; echocardiography may optimize resuscitation
Leviter et al. [14] (2023)	Manual pulse check vs. POCUS (cardiac and femoral)	Manual: unreliable; POCUS: detected cardiac activity and confirmed IO flow	POCUS: rapid, real-time	NR	Manual pulse missed in both cases; POCUS led to timely post-ROSC care and confirmation of effective compressions/IO infusion
Yanni et al. [15] (2023)	POCUS for cardiac standstill	Implied acceptable accuracy among experienced PEM attendings	6 s per video clip (clip duration)	Krippendorff’s α (Kα)=0.740 (95% CI 0.735-0.745) for PEM attendings with ≥25 scans; Kα=0.304 (95% CI 0.287-0.321) for clips with wall motion without valve motion	Potential misinterpretation when wall motion occurs without valve motion; suboptimal views; lack of formal reference standard affecting agreement

Quality Assessment on QUADAS-2 Tool

The methodological quality of the six included studies, as assessed by the QUADAS-2 tool, was variable. The studies by Tibballs and Russell [11], Tibballs and Weeranatna [12], Tsung and Blaivas [13], and Yanni et al. [15] were judged to have a low risk of bias across all four domains (patient selection, index test, reference standard, and flow and timing) and low concerns regarding their applicability to the review question. In contrast, the simulation study by Inagawa et al. [10] was rated as having a high risk of bias in the index test, reference standard, and flow and timing domains, and high concerns regarding applicability in all domains, primarily due to its manikin-based design, which limits generalizability to clinical practice. Similarly, the case series by Leviter et al. [14] was assessed as having a high risk of bias in the index test, reference standard, and flow and timing domains, with high concerns regarding the applicability of the reference standard, reflecting the inherent limitations of a small, illustrative case report in providing generalizable evidence on diagnostic accuracy (Table 3).

**Table 3:** Quality assessment of included studies using the QUADAS-2 tool QUADAS-2, Quality Assessment of Diagnostic Accuracy Studies - Version 2

Study (author and year)	Risk of bias				Applicability concerns		
	Patient selection	Index test	Reference standard	Flow and timing	Patient selection	Index test	Reference standard
Inagawa et al. [10] (2003)	Low	High	High	High	High	High	High
Tibballs and Russell [11] (2009)	Low	Low	Low	Low	Low	Low	Low
Tibballs and Weeranatna [12] (2010)	Low	Low	Low	Low	Low	Low	Low
Tsung and Blaivas [13] (2008)	Low	Low	Low	Low	Low	Low	Low
Leviter et al. [14] (2023)	Low	High	High	High	Low	High	High
Yanni et al. [15] (2023)	Low	Low	Low	Low	Low	Low	Low

Discussion

Main Findings

This systematic review evaluated the reliability of manual pulse checks against emerging techniques, primarily POCUS, in the context of pediatric resuscitation. By synthesizing evidence from six heterogeneous studies, our findings paint a consistent picture: traditional manual pulse palpation is an inherently unreliable method for diagnosing cardiac arrest in children, characterized by variable accuracy, slow decision-making, and a high propensity for critical errors. In contrast, emerging techniques, particularly POCUS, demonstrate a promising potential to overcome these limitations by providing objective, real-time visual confirmation of cardiac activity, albeit within an evolving evidence base that currently includes simulation studies and preliminary clinical observations. The convergence of these results across different study designs and settings underscores a critical juncture in pediatric resuscitation science, where the longstanding reliance on pulse palpation is being challenged by technological advancements that could fundamentally enhance the precision and efficacy of life-saving interventions.

Clinical Implications

The unreliability of manual pulse palpation, as documented in this review, is alarming yet consistent with a broader body of literature. The studies by Tibballs and colleagues [11,12] provide compelling evidence that even among healthcare professionals, the sensitivity and specificity of pulse checks are suboptimal, leading to unacceptable rates of both withheld and unnecessary chest compressions. These findings resonate with adult resuscitation studies. For instance, Eberle et al. [16], in a seminal study on pulse check accuracy in adult cardiopulmonary resuscitation (CPR), found that carotid pulse checks by healthcare providers had a sensitivity of only 90% and a specificity of 55%, meaning that nearly half of the patients with a pulse might have had compressions incorrectly initiated. Similarly, Lapostolle et al. [17] demonstrated that even emergency physicians could not reliably determine pulselessness within 10 seconds in adult patients. The pediatric context, however, introduces unique challenges, such as smaller vessel size, higher heart rates, and the heightened emotional stress of managing a critically ill child, which may further degrade the performance of manual palpation. The work of Inagawa et al. [10] in a simulated infant environment, while limited in applicability, starkly illustrates the technical difficulty, with femoral pulse detection accuracy plummeting to 42.9%. This suggests that the problem is not merely one of training or haste but is fundamentally rooted in the physiological and practical limitations of tactile perception under high-stress conditions. The consequence, as shown by Tibballs and Russell [11], is a tangible clinical impact: a misdiagnosis rate of 22% directly translates to delays in definitive care and interruptions in high-quality CPR, which are known to adversely affect survival outcomes.

The promise of POCUS, as illustrated in this review, lies in its ability to bypass the subjective nature of palpation. The cases presented by Leviter et al. [14] are instructive; POCUS not only correctly identified cardiac activity missed by manual checks but also provided additional diagnostic and procedural guidance, such as confirming IO flow. This multifunctional utility aligns with a growing consensus in adult emergency medicine. Breitkreutz et al. [18] were among the first to systematically propose the concept of "Focused Echocardiographic Evaluation in Resuscitation" (FEER), demonstrating that brief, targeted ultrasound exams could rapidly identify reversible causes of cardiac arrest and guide therapy. Furthermore, a study by Blyth et al. [19] showed that echocardiography during adult cardiac arrest could predict survival to hospital admission, with the presence of cardiac activity on ultrasound being a strong positive predictor. The quantitative inter-rater reliability data from Yanni et al. [15] are particularly significant. A Krippendorff’s alpha of 0.740 among experienced PEM physicians for identifying cardiac standstill suggests a level of diagnostic consistency that is unattainable with manual palpation. This echoes findings in adult cardiac arrest, where Gaspari et al. [20] found excellent agreement (kappa = 0.93) among emergency physicians for interpreting cardiac motion on POCUS. The rapid interpretation time of 6 seconds per clip reported by Yanni et al. [15] is crucial, as it addresses the primary concern that ultrasound may prolong pulse check intervals beyond the American Heart Association's recommended 10-second limit. This finding is supported by work in simulated adult scenarios, such as that by In't Veld et al. [21], which demonstrated that trained clinicians could perform a cardiac POCUS exam during a standardized pulse check with minimal disruption to CPR rhythm.

Comparison With Existing Literature

However, the integration of POCUS into routine pediatric resuscitation is not without significant challenges, a nuance that must be acknowledged when interpreting our synthesized findings. The evidence base, as captured in this review, remains nascent. The studies by Tsung and Blaivas [13] and Leviter et al. [14] are preliminary, comprising small case series that, while illustrative, lack the statistical power to make broad generalizations. The simulation-based nature of the Yanni et al. [15] study, though methodologically robust for assessing agreement, does not fully capture the chaos and technical difficulties of a real-world resuscitation, where obtaining optimal subxiphoid or parasternal views can be hampered by patient body habitus, ongoing compressions, and clinical clutter. The identified challenge of interpreting subtle wall motion without valve activity [15] is a critical learning point; it highlights the need for sophisticated training that goes beyond simple "cardiac activity versus standstill" and delves into the nuances of pseudo-pulseless electrical activity (PEA). This is consistent with concerns raised in the adult literature. Clattenburg et al. [22] noted that the presence of any cardiac movement on POCUS, even without corresponding output, could lead to confusion about whether to continue compressions, a scenario termed "POCUS-confirmed PEA." This necessitates clear protocols and advanced interpreter competency to avoid misinterpretation that could paradoxically harm the patient. Moreover, the resource implications, cost of equipment, training requirements, and maintenance of skills are substantial barriers to widespread implementation, particularly in resource-limited settings where the burden of pediatric mortality is highest. The learning curve for POCUS is not trivial, and the expertise demonstrated by the PEM attendings in Yanni et al. [15] (who had performed ≥25 scans) may not be generalizable to all frontline providers.

When placed in the context of the existing literature, the findings of this review both confirm and extend previous knowledge. Our results strongly corroborate the conclusions of a prior systematic review by Katzenschlager et al. [9] on the use of POCUS in pediatric cardiac arrest, which also found that ultrasound was feasible and more accurate than clinical assessment alone. However, our review adds a more granular understanding of the specific performance metrics of manual palpation from the Tibballs studies [11,12] and provides newer data on inter-rater reliability from Yanni et al. [15]. Conversely, our findings contrast with a more conservative viewpoint expressed in some older guidelines, which, in the absence of robust pediatric evidence, have been hesitant to endorse POCUS for fear of causing delays. The data presented here, particularly the swift interpretation times [10,15], suggest that this concern can be mitigated with adequate training. Furthermore, while the review by Chen et al. [23] focused on the use of ultrasound to guide fluid therapy in shock, it indirectly supports our findings by demonstrating that POCUS can rapidly provide objective hemodynamic information that supersedes unreliable clinical signs, a principle that is directly transferable to the arrest state. The trajectory of the evidence suggests a paradigm shift akin to the adoption of waveform capnography for confirming endotracheal tube placement and measuring CPR quality; what was once an advanced tool is now considered a standard of care. POCUS for pulse checks may be following a similar path from novel innovation to essential diagnostic tool.

Limitations

This systematic review has several important limitations that must be considered when interpreting its conclusions. First, the small number of included studies (n=6) and their significant methodological heterogeneity, encompassing simulation, in-hospital studies on specialized populations, and case series, limit the strength of any meta-analytic conclusions and preclude a formal quantitative synthesis. The overall quality of the evidence, as assessed by the QUADAS-2 tool, was variable, with simulation studies [10,12] and case reports [14] carrying a high risk of bias and limited applicability to general clinical practice. The search strategy, while comprehensive, may not have captured all relevant grey literature or studies in non-English languages, potentially introducing selection bias. Furthermore, the rapid evolution of ultrasound technology and its increasing accessibility mean that the findings of older studies may not fully reflect current capabilities and training standards. Finally, the review was unable to address the critical outcome of patient-centered endpoints, such as survival to hospital discharge or neurological outcome, as the included studies primarily reported on diagnostic accuracy and process measures. The ultimate impact of POCUS on pediatric arrest survival remains an urgent question for future research.

## Conclusions

Manual pulse palpation is an unreliable method for diagnosing cardiac arrest in pediatric patients, often leading to diagnostic errors with direct clinical consequences. The emerging technique of POCUS presents a compelling alternative, offering a more objective, accurate, and rapid means of assessing cardiac activity, with the added benefit of providing guidance for resuscitation management. While challenges related to training, interpretation, and resource allocation remain, the consistent direction of the evidence points toward the potential for POCUS to significantly enhance the quality of care during pediatric resuscitation. Future research should prioritize large, prospective RCTs conducted in real-world settings to definitively establish the impact of POCUS-guided pulse checks on survival and neurological outcomes, thereby informing the next generation of evidence-based resuscitation guidelines.

## Appendices

**Table 4:** Search strategy for included databases

Database	Search date	Search strategy	Results (n)
PubMed/MEDLINE	September 02, 2025	((“pulse check”[Title/Abstract] OR “pulse palpation”[Title/Abstract] OR “manual pulse”[Title/Abstract] OR “pulse assessment”[Title/Abstract]) AND (“pediatric”[Title/Abstract] OR “paediatric”[Title/Abstract] OR “child*”[Title/Abstract] OR “infant*”[Title/Abstract]) AND (“resuscitation”[Title/Abstract] OR “cardiac arrest”[Title/Abstract] OR “CPR”[Title/Abstract]) AND (“point-of-care ultrasound”[Title/Abstract] OR “POCUS”[Title/Abstract] OR “ultrasound”[Title/Abstract] OR “echocardiography”[Title/Abstract] OR “near-infrared spectroscopy”[Title/Abstract] OR “NIRS”[Title/Abstract] OR “capnography”[Title/Abstract] OR “emerging technolog*”[Title/Abstract]))	83
Web of Science (Core Collection)	September 01, 2025	TS=(("pulse check" OR "manual pulse" OR "pulse palpation" OR "pulse assessment") AND ("pediatric" OR "paediatric" OR child* OR infant*) AND ("resuscitation" OR "cardiac arrest" OR CPR) AND ("point-of-care ultrasound" OR POCUS OR echocardiography OR ultrasound OR "near-infrared spectroscopy" OR NIRS OR capnography OR "emerging technolog*"))	56
Scopus (Elsevier)	September 02, 2025	TITLE-ABS-KEY(("pulse check" OR "manual pulse" OR "pulse palpation" OR "pulse assessment") AND ("pediatric" OR "paediatric" OR child* OR infant*) AND ("resuscitation" OR "cardiac arrest" OR CPR) AND ("point-of-care ultrasound" OR POCUS OR echocardiography OR ultrasound OR "near-infrared spectroscopy" OR NIRS OR capnography OR "emerging technolog*"))	62
Embase (Elsevier)	September 01, 2025	(‘pulse check’:ab,ti OR ‘pulse palpation’:ab,ti OR ‘manual pulse’:ab,ti OR ‘pulse assessment’:ab,ti) AND (‘pediatric’:ab,ti OR ‘paediatric’:ab,ti OR child*:ab,ti OR infant*:ab,ti) AND (‘resuscitation’:ab,ti OR ‘cardiac arrest’:ab,ti OR ‘CPR’:ab,ti) AND (‘point-of-care ultrasound’:ab,ti OR ‘POCUS’:ab,ti OR ‘echocardiography’:ab,ti OR ‘ultrasound’:ab,ti OR ‘near-infrared spectroscopy’:ab,ti OR ‘NIRS’:ab,ti OR ‘capnography’:ab,ti OR ‘emerging technolog*’:ab,ti)	39
